# Treatment Strategies for High-Risk Localized and Locally Advanced and Oligometastatic Prostate Cancer

**DOI:** 10.3390/cancers13174470

**Published:** 2021-09-05

**Authors:** Tomoyuki Makino, Kouji Izumi, Hiroaki Iwamoto, Atsushi Mizokami

**Affiliations:** 1 Department of Integrative Cancer Therapy and Urology, Kanazawa University Graduate School of Medical Science, 13-1 Takara-machi, Kanazawa 920-8640, Ishikawa, Japan; mackeeen511@gmail.com (T.M.); iwamoto-h@med.kanazawa-u.ac.jp (H.I.); mizokami@staff.kanazawa-u.ac.jp (A.M.); 2 Department of Urology, Ishikawa Prefectural Central Hospital, Kanazawa 920-8530, Ishikawa, Japan

**Keywords:** prostate cancer, locally advanced, oligometastasis, very high-risk

## Abstract

**Simple Summary:**

The definitions of locally advanced and oligometastatic prostate cancer are ambiguous, and there are no standard treatments for these. Although multidisciplinary treatment combining systemic and local treatment may be effective, there are many unresolved issues such as the choice of local treatment, use of new endocrine agents and chemotherapy, and selection of optimal patients. The present article discusses the definitions, diagnoses, and treatment of very high-risk prostate cancer and oligometastatic prostate cancer.

**Abstract:**

Despite the significant advances in the treatment of high-risk prostate cancer, patients with very high-risk features such as being locally advanced (clinical stage T3–4 or minimal nodal involvement), having a high Gleason pattern, or with oligometastasis may still have a poor prognosis despite aggressive treatment. Multidisciplinary treatment with both local and systemic therapies is thought to be effective, however, unfortunately, there is still no standard treatment. However, in recent years, local definitive therapy using a combination of radiotherapy and androgen deprivation is being supported by several randomized clinical trials. This study reviews the current literature with a focus on the definition of very high-risk prostate cancer, the role of modern imaging, and its treatment options.

## 1. Introduction

With the introduction of prostate cancer (PC) screening, more men are continuously being diagnosed with clinically nonmetastatic PC. However, 17–31% of them have high-risk localized or locally advanced disease requiring curative treatment [1]. In addition, with the development of new imaging techniques, several types of oligometastatic PC which are between locally advanced cancer and widely metastatic cancer are being discovered [2]. Thus, accurate diagnosis will become more important in the future. Recent advancements have also been made in radiotherapy (RT) for PC due to innovations in medical and physical engineering, and excellent long-term results have been reported [3,4,5,6,7,8,9]. However, the definitions of locally advanced PC and oligometastatic PC are still ambiguous, and there is no standard treatment for these. Furthermore, no studies directly compare local treatment options such as radical prostatectomy (RP) and RT. Thus, the superiority or inferiority of these treatments is unknown. So far, the standard treatment for oligometastatic PC has been systemic therapy, specifically androgen deprivation therapy (ADT). In recent years, however, the significance of combined local treatment has been discussed, and the results of large-scale clinical trials have been reported with and without combined RT [6,10]. In addition, attempts to further enhance disease control by concomitant metastasis-directed therapy (MDT) have been made [11,12,13], and radical curing of oligometastatic PC may be expected in the future. The present article reviews the definition, diagnosis, and treatment (especially RT) of locally advanced (focusing on very high-risk PC) and oligometastatic PC, focusing on previous reports.

## 2. Locally Advanced PC

### 2.1. Definition

Since D’Amico et al. proposed the risk classification of localized PC, risk stratification has been conducted by various research groups and guidelines. Cases with a high risk of recurrence are defined as “high-risk PC” [14]; however, because of the heterogeneity in prognosis among these cases, they have been further subdivided into “very high-risk PC” [15]. Sphan et al. studied 712 PC patients with prostate-specific antigen (PSA) > 20 ng/mL and found that a combination of additional risk factors (e.g., Gleason score (GS) 8–10, clinical stage (c) T3–4) at presentation were associated with unfavorable histopathology and worse cancer-specific outcomes [16]. Similarly, Walz and Joniau showed that patients with two or more high-risk features (e.g., PSA > 20 ng/mL, GS 8–10, cT3–4) had worse biochemical recurrence-free survival (RFS) and prostate cancer-specific survival (CSS) than those with only one high-risk feature [17,18]. In contrast, the preoperative criteria of the National Comprehensive Cancer Network (NCCN) identifies those with primary Gleason pattern 5 on biopsy or ≥4 cores containing pattern 4 (odds ratio (OR): 3.17; *p* < 0.001) as the high-risk subgroup, who are most likely to experience early (within one year) biochemical recurrence after surgery [19]. In recent years, the NCCN defined very high-risk PC patients as those with cT3b–4 or primary Gleason pattern 5 or >4 cores with grade group ≥ 4 according to the International Society of Urological Pathology [20].

Lymph node involvement was considered as a sign of further metastases indicating poor prognosis. However, low nodal metastatic burden in surgical specimens indicates better outcomes than visceral or bone metastasis. Patients with ≤2 positive lymph nodes treated with RP with extended pelvic lymph node dissection show significantly better CSS outcomes at 15-year follow-up compared to those with ≥2 positive lymph nodes (84% vs. 62%, respectively; *p* < 0.001) [21]. Moreover, patients with a GS < 8 and ≤2 positive lymph nodes had relatively favorable outcomes at 10 years among patients with lymph node metastases treated with RP and extended pelvic lymphadenectomy [22]. These findings suggest that the current staging system of PC does not accurately reflect prognosis and treatment paradigms may need to be reconsidered for better outcomes. Indeed, the recent European Association of Urology (EAU) guidelines state that cN+ patients are categorized as having high-risk locally advanced disease; and that cT3–4 or cN+ (any PSA and any GS) cases can be ultimately managed locally through a multimodal approach [23]. The definitions of very high-risk PC reported so far are shown in Table 1.

### 2.2. Diagnosis

Imaging studies are very important in the diagnosis of locally advanced PC. A cohort study of high-risk PC using multi-parametric magnetic resonance imaging (mpMRI) with a combination of T2-weighted, diffusion-weighted, and dynamic contrast-enhanced images reported an 89% positivity rate of extracapsular invasion and an OR of 10.3 for predicting extracapsular invasion [24]. In addition, a meta-analysis including 17 studies that used mpMRI to detect extracapsular invasion reported a sensitivity of 0.55 (95% confidence interval (CI): 0.43–0.66) and a specificity of 0.87 (95% CI: 0.82–0.91), suggesting the usefulness of mpMRI for diagnosing locally advanced PC [25]. The NCCN, EAU, and American Urological Association guidelines recommend mpMRI for the diagnosis of locally advanced PC [20,23,26], and it is expected to become more widely used in the future.

### 2.3. Treatment

Previously, ADT alone has been the most common treatment for very high-risk PC. Although combining systemic treatment with ADT and local treatment has recently become more common, there is no standardized treatment for the choice of local treatment because it is unknown which is better between RP and RT; no direct comparison studies have been conducted. Recently, RP plus RT and RT plus ADT in patients with T3-4N0-1M0 PC were compared and PC-specific survival rates for patients undergoing RP plus RT and RT plus ADT, respectively, were 88.9% and 74.2% for T3a/bN0-NXM0 disease, 75.7% and 58.6% for T3a/bN1M0 disease, and 72.0% and 60.5% for T4N0–NXM0 disease. [27]. A retrospective analysis was also performed in 2935 elderly (≥65 years) males in the Surveillance Epidemiology and End Results (SEER)-Medicare linked database who underwent external beam RT (EBRT) versus RP for locally advanced PC [28]. EBRT was associated with higher overall and prostate-specific mortality (hazard ratio (HR): 1.41; 95% CI: 1.09–1.82 vs. HR: 2.35; 95% CI: 1.85–2.98, respectively). Although RP-treated patients appeared to have better prognoses than RT-treated ones, these studies are not prospective comparative studies, and the method and dose of irradiation are unknown. Thus, caution should be taken in interpreting these results, and definitive conclusions cannot be drawn.

#### 2.3.1. RP

In most high-risk patients, surgery is performed with the goal of cure. However, for clinical T3b–4 PC, RP can be thought of as “debulking” the primary tumor in order to improve local control. Moltzahn et al. evaluated a multicenter cohort of 266 patients with very high-risk locally advanced PC (cT3b–4) treated surgically. Despite poor pathologic characteristics, the 10-year cancer-specific mortality rate was relatively low (5.6–12.9%) and was influenced by comorbidities and age [29]. In a report based on the SEER database including 1093 patients with cT4 or cN1 PC, the five-year overall survival (OS) of patients treated with RP or combined ADT and RT was significantly better than that of patients treated with ADT alone or without treatment [30]. The long-term outcome of PC patients who underwent RP in a European cohort has also been reported [31]; this assessed the 20-year oncological outcomes of 22,843 patients, of which there were 3230 high-risk and 903 very high-risk patients based on the NCCN classification. High-risk patients had a 20-year biochemical RFS rate of 34.5%, whereas very high-risk patients had the steepest decline in biochemical RFS and did not reach the 20-year follow-up (30.5% at 15 years). The 20-year metastasis-free survival (MFS) of the high- and very high-risk groups were 64.8% and 64.1%, respectively. The 20-year CSS in the high- and very high-risk groups were 69.6% and 60.8%, respectively. In recent years, robot-assisted RP (RARP) has rapidly become popular, and there has been an accumulating number of reports with good results comparable to those of conventional open surgery. In a recent report, the three-year RFS of RARP plus extended pelvic lymph node dissection for locally advanced PC of cT3 or higher was 95.8% [32].

#### 2.3.2. Radiation Therapy

The first large comprehensive study comparing long-term follow-up of risk-stratified patients for each treatment option has been reported [33]. Among them, brachytherapy with EBRT plus ADT, as well as high-dose-rate (HDR) brachytherapy were reported as treatment options with relatively good results for high-risk patients. On the other hand, in a randomized phase 3 trial of 1205 patients with locally advanced PC staged at cT3-4N0/NXM0, cT1–2 + PSA > 40 ng/mL, or cT1–2 + PSA 20–40 ng/mL + GS 8–10, the 10-year OS of patients treated with combined ADT and RT was significantly better than that of patients treated with ADT alone, at 55% and 49%, respectively (HR: 0.70; 95% CI: 0.57–0.85; *p* < 0.001), and the risk of death was reduced by 30% [5]. Furthermore, many large studies have demonstrated that ADT plus RT was more effective than ADT alone [34,35,36,37]. The prospective randomized trial RTOG 85-31 evaluated the effectiveness of adjuvant ADT after definitive RT (a total dose of 65–70 Gy including a boost to the prostate) in patients with cT3 and/or regional lymph node involvement and showed the addition of ADT significantly improved 10-year OS (49% vs. 39%, *p* = 0.002) [3,4]. The GETUG 12 trial evaluated the addition of docetaxel and estramustine to adjuvant ADT in RT or RP among patients with high-risk localized PC and showed that the combined group significantly improved the eight-year RFS (62% vs. 50%, *p* = 0.017) [7].

Although local treatment of PC in men with pelvic lymph node metastasis was considered futile, the advantage of combining ADT with RT may be a promising treatment option. Rusthoven et al. reported that additional EBRT for prostate to ADT improved the 10-year estimated CSS (67% and 53%, respectively; *p* < 0.001) in 796 patients with cT1-4N1M0 PC [8]. Evaluation of 3540 patients with clinically lymph node positive PC from the National Cancer Data Base also reported treatment with ADT plus RT was associated with a 50% reduction in five-year all-cause mortality when compared to treatment with ADT alone (HR: 0.5, 95% CI: 0.37–0.67; *p* < 0.001) [9]. Moreover, the duration of ADT was reported to be superior in terms of local control and prognosis with long-term treatment (24–36 months) compared with short-term treatment (≤6 months) [36]. In addition, in the case of very high-risk PC, potential micrometastases may be present, and hormonal therapy is expected to have a therapeutic effect on these. Thus, these findings establish the significance of combined long-term ADT and RT for very high-risk PC.

As for the significance of combining RT and hormonal therapy, there may be additive and synergistic effects due to the possibility of enhanced apoptosis. Zitman et al. reported that Shionogi tumors can be transplanted into mice and that castration may sensitize them to the cell-killing effects of radiation [38]. In addition, Pollack et al. reported that castration and fractionated irradiation significantly amplified the growth retardation effect in rat PC cells [39].

The options of RT have since diversified to include three-dimensional conformal RT (3D-CRT), intensity-modulated RT, volumetric-modulated arc therapy, stereotactic body RT, and brachytherapy, specifically permanent low-dose-rate (LDR) brachytherapy and temporary HDR brachytherapy. However, it is unclear which irradiation method is optimal so far. Although the therapeutic effect differs depending on the irradiation method, an index called the biologically effective dose (BED) is a common indicator for judging the effectiveness, with higher BED indicating more effective treatment. Stone and Stock et al. reported that the risk of PSA recurrence decreases with increasing BED in brachytherapy with or without EBRT treatment [40]. A recent meta-analysis on appropriate irradiation doses for PC by Zaorsky et al. reported that an increase in BED to 200 Gy was associated with increased PC control, however, doses above 200 Gy did not result in additional clinical benefit [41].

The α/β value of PC cells is an index of their responsiveness to irradiation. This was analyzed and reported to be 1.5 Gy, which is much lower than that of normal tumor cells [42,43]. This is lower than the normal tissue α/β value of 3 Gy. Theoretically, this finding suggests that hypofractionated irradiation with an increased dose per line can safely increase the dose to the prostate without increasing the frequency of late adverse events in normal tissues. Although hypofractionated irradiation has been tested in various irradiation methods and its therapeutic results are being reported, there are still few reports for high- and very high-risk PC.

- Three-dimensional conformal radiotherapy

The most widespread method of external irradiation is 3D-CRT. In this method, the irradiation area is adjusted according to the shape of the target affected area, so that normal tissues are, as much as possible, not exposed to radiation. It has been reported that 3D-CRT for high-risk localized or locally advanced PC is associated with excellent tumor control and survival outcomes [44,45,46]. Zelefsky et al. have shown that the 10-year PSA relapse-free survival outcomes for stage T3a and T3b tumors were 44% and 32%, respectively, whereas the 10-year CSS outcomes for stage T3a and T3b tumors were 88% and 79%, respectively [44].

- Intensity-modulated radiotherapy

Intensity-modulated radiotherapy (IMRT) is an evolved form of 3D-CRT which provides optimal dose distribution to lesions that are close to risk organs or have complex shapes by irradiating these with a radiation beam with spatially and temporally non-uniform intensity from multiple directions. Currently, IMRT is the standard form of EBRT for PC, rather than 3D-CRT, because of its ability to reduce gastrointestinal and genitourinary (GU) toxicities, biochemical recurrence, and mortality [47,48,49]. In addition, IMRT may also be used to irradiate pelvic lymph nodes in cases of high-risk or locally advanced PC. A well-conducted, non-randomized, propensity-matched retrospective analysis of 42,481 patients in the U.S. National Cancer Database found IMRT to be beneficial in intermediate-risk (*p* < 0.001) and high-risk PC (*p* < 0.001), but not in low-risk PC (*p* = 0.54) [50]. Moreover, the results of high-dose RT (78 Gy in 92.5% of patients) with IMRT for cT3–4 PC showed eight-year RFS, CSS, and OS rates of 53.2% (95% CI: 43.4–62.1%), 96.6% (95% CI: 91.2–98.7%), and 89.1% (95% CI: 81.5–93.7%), respectively, at a median observation period of 97 months without grade 4 or higher side effects [51]. Wilcox et al. reported the mid-term results of modern EBRT, using combined ADT and dose-escalated IMRT (78 Gy) with MRI-computed tomography (CT) fusion and daily image guidance with fiducial markers. High-risk PC patients had five-year biochemical RFS and CSS rates of 91.3% and 97.7%, respectively, and late rectal adverse event rates of 1.6% for grade 2 and 0% for grade 3, after a median follow-up of 59 months [52]. In an analysis of IMRT for T3b PC, Goupy et al. found the five-year risks of biochemical recurrence, clinical recurrence, and cancer-specific death to be 24.8%, 21.7%, and 10.3%, respectively [53]. On the other hand, there have been few trials of the aforementioned hypofractionated irradiation in high-risk PC. In a multicenter phase 3 trial, the Hypofractionated Irradiation for Prostate Cancer (HYPRO) trial, hypofractionated RT (64.6 Gy; 19 fractions of 3–4 Gy, three fractions per week) was compared with conventionally fractionated RT (78 Gy; 39 fractions of 2 Gy, five fractions per week) for the treatment with IMRT of T1b-4N0/NXM0/MX PC, including 74% high-risk PC patients [54]. This study reported that the five-year relapse-free survival was 80.5% (95% CI: 75.7–84.4%) for patients treated with hypofractionation and 77.1% (95% CI: 71.9–81.5%) for those treated with conventional fractionation (adjusted HR: 0.86; 95% CI: 0.63–1.16; log-rank *p* = 0.36). A phase 2 study of hypofractionated IMRT (70 Gy/28 fractions) using image-guided radiation techniques for PC, including high-risk, is currently underway (UMIN000007810), and the report on its results is awaited.

- Volumetric-modulated arc therapy

Volumetric-modulated arc therapy (VMAT) is a new irradiation method, combining conventional IMRT and rotational irradiation technology to shorten the treatment time. This has resulted in a reduction in patient burden as well as a further improvement in irradiation accuracy. Although the clinical use of VMAT is increasing significantly worldwide, most currently published data are limited to planning and feasibility studies [55]. Results relative to toxicity and clinical outcome are emerging, but are still sparse, and there are few reports in PC treatment. One such study by Hegazy et al. reported that the three-year biochemical RFS rates for high-risk PC were 48% at a median follow-up period of 34 months [56]. Therefore, larger trials with longer follow-up periods are needed in the future.

- Stereotactic body radiotherapy

Stereotactic body radiotherapy (SBRT) is an advanced conformal RT technique that allows for ultra-hypofractionation, thereby significantly reducing the overall treatment time [57,58]. Available studies have convincingly shown that SBRT can be performed safely and with excellent results in patients with low- and intermediate-risk PC [59,60,61]. However, the role of SBRT in patients with high- and very high-risk PC remains unclear, as only a few patients were included in the clinical trials conducted [59]. A recent systematic report showed that biochemical control rates ranged from 82–100% at two years and 56–100% at three years, with only a few studies reporting longer-term follow-up data [62]. Thus, with the currently available evidence, it can be concluded that SBRT with or without pelvic elective nodal irradiation cannot be considered the standard of care in high-risk PC due to a lack of high-level evidence [62].

- LDR and HDR brachytherapy

Brachytherapy is a type of RT in which a sealed radiation source is placed directly into the body [63]. The placement of the radiation source into the prostate can be permanent or temporary. In permanent interstitial brachytherapy, also known as seed brachytherapy, an LDR radioactive source is placed in the prostate and left permanently to gradually release radiation over time. In temporary brachytherapy, a needle or catheter is first placed in the prostate to confirm its exact location, and then a radioactive source is temporarily introduced into the prostate. The radiation is delivered using a HDR device and the actual treatment time lasts a few minutes.

Although LDR brachytherapy is indicated for patients with low-risk PC and low volume intermediate-risk PC, there is a significant risk of extracapsular diffusion that is not included in the high-dose region of seed implantation in high-risk patients [63]. In such cases, brachytherapy may be combined with EBRT to ensure that appropriate targets are treated. The ASCENDE-RT study is a randomized comparison of two methods of dose escalation in the context of combined modality therapy for high- and intermediate-risk PC (NCCN classification) that includes 12 months of ADT and whole pelvic irradiation to 46 Gy compared with ^125^I brachytherapy and EBRT boost to a total of 78 Gy. This study found that the LDR boost improved PSA control compared to EBRT alone, but at the cost of higher GU late toxicity [64]. In recent years, there has been an expansion of the indications for sealed small source therapy, which allows for high-dose local irradiation, and good results have been reported in high-risk PC patients, especially for trimodal therapy (a combination of LDR brachytherapy, EBRT, and hormonal therapy) [65,66]. Moreover, a phase 3 multicenter randomized controlled trial (UMIN000003992) investigating trimodal therapy (with LDR brachytherapy, EBRT, and hormonal therapy) for high-risk PC is currently underway, and future results are highly anticipated [67].

With respect to radiobiology, the degree of dose increase that can be achieved with HDR brachytherapy may be more effective in killing PC cells when compared to other EBRT techniques and LDR brachytherapy [68]. Thus, the current clinical evidence for equivalent outcomes with either LDR and HDR or HDR brachytherapy having a theoretical advantage was supported by radiobiological models. There are no other prospective randomized comparisons of EBRT and HDR boost, although some single institution reports have demonstrated favorable biochemical control rates for high-risk localized and locally advanced PC [69,70,71,72,73,74]. Yamazaki et al. have shown the efficacy of dose escalating RT in patients with cT3b–4 PC. The biochemical disease-free rate at five years was 78.9% (95% CI: 69.8–87.9%) in the EBRT and HDR boost group compared to 66.5% (95% CI, 56.1–76.9%) in the conventional EBRT group treated with 70–72 Gy [73]. Moreover, Kasahara et al. reported the outcomes of HDR brachytherapy combined with hypofractionated EBRT in 66 patients classified as very high-risk PC (NCCN classification) [74]. The five-year biochemical failure-free, distant metastasis-free, PC-specific, and OS rates were 88.7%, 89.2%, 98.5%, and 97.0%, respectively. However, all these studies are retrospective, with small cohort sizes and short follow-up periods. Kishan et al. recently reported a retrospective multicenter study analyzing RP, EBRT, and EBRT with a brachytherapy boost (LDR in 262 patients, HDR in 174 patients) in 1809 patients with GS 9–10 PC [75]. The five-year MFS rates were 76% for RP, 76% for EBRT, and 92% for EBRT plus brachytherapy, which was associated with a significantly lower rate of distant metastasis (HR: 0.27 (95% CI: 0.17–0.43) for RP and 0.30 (95% CI: 0.19–0.47) for EBRT). The 7.5-year OS rates were 83% for RP, 82% for EBRT, and 90% for EBRT plus brachytherapy, which was associated with significantly lower all-cause mortality (HR: 0.66 (95% CI: 0.46–0.96) for RP and 0.61 (95% CI: 0.45–0.84) for EBRT). The superiority of brachytherapy boost over both RP and EBRT was clearly shown by these results. Interestingly, EBRT with brachytherapy potentially offers improved local control over EBRT, which may prevent subsequent metastases.

Incidentally, the BED achieved with HDR brachytherapy boost is typically much higher than can be achieved with EBRT alone. At an α/β ratio of 1.5, the BEDs were 200–300 Gy for HDR brachytherapy versus −187 Gy for EBRT monotherapy (80 Gy in 2 Gy fractions) [76]. In contrast, the combination of EBRT at a dose of 45 Gy with a permanent implant of ^125^I with a D90 (minimal dose received by 90% of the target volume) ≥130 Gy achieved a BED of >220 Gy, using an α/β ratio of 2 [40,77]. Moreover, conventionally fractionated RT with or without dose-escalated and hypofractionated RT reached a BED of 140–200 Gy using an α/β ratio of 1.5 [41]. Thus, from the BED perspective, an HDR boost may be more efficient.

Table 2 shows the results of various treatments for high-risk localized and very high-risk PC that have been reported so far.

## 3. Oligometastatic PC

### 3.1. Definition

Oligometastasis is a concept first proposed by Hellman and Weichselbaum in 1995 [78]. It is considered as a metastatic carcinoma that lies between locally advanced carcinoma and widely metastatic carcinoma, and it should be treated separately from both in terms of prognosis and treatment [2]. Although oligometastasis is usually defined as a limited number of clinically detectable metastases, it has no formally established definition. The recently reported definitions of oligometastatic PC are shown in Table 3. There are many definitions of metastatic sites, ranging from bone and lymph nodes to any site, and the number of metastases is often reported to be three to five or less [79,80,81,82,83,84,85]. Basically, these disease states are not special biologically and pathologically, but are just in between non-visualized and bulky metastatic disease, and may be treated as early/low metastatic disease.

### 3.2. Diagnosis

In addition to conventional imaging methods using CT and bone scintigraphy, many reports have used positron emission tomography-CT (PET-CT) using ^18^F-fluorodeoxyglucose and ^11^C-choline, as well as whole-body MRI (WB-MRI) for diagnosis. Shen et al. conducted a meta-analysis of the diagnostic performance of bone metastases and reported that choline PET-CT, MRI, and bone scintigraphy had sensitivities of 87%, 95%, and 79%, respectively, and specificities of 97%, 96%, and 82%, respectively [86]. Recently, PET using prostate-specific membrane antigen (PSMA), a type II transmembrane protein highly expressed in PC, as a tracer has been attracting attention as a promising new imaging modality [87,88]. Maurer et al. reported that ^68^Ga-PMSA-PET had a sensitivity of 66% and a specificity of 99% for the diagnosis of lymph node metastasis [89]. In addition, a meta-analysis including 1309 patients reported that the positivity rates of ^68^Ga-PSMA-PET for patients with biochemical recurrence were 42%, 58%, 76%, and 95% for PSA categories 0–0.2, 0.2–1, 1–2, and >2 ng/mL, respectively [90]. Thus, PSMA-PET has improved specificity and sensitivity compared to standard imaging (CT, MRI, and bone scintigraphy), and also improves the detection of metastatic disease in biochemically recurrent PC with low serum PSA levels. Therefore, it is expected to reliably diagnose clinical stage, which would lead to early intervention in cases of locally advanced cancer with potential metastasis and in more extensive metastatic cancer, even in oligometastatic PC. This also allows for the accurate detection of metastatic sites in biochemically recurrent cases. Thus, PSMA PET/CT, which has relatively high sensitivity and specificity compared to other imaging modalities, is increasingly being used for staging and defining oligometastatic PC. Although there are currently no randomized data showing better clinical outcomes with the use of PSMA PET/CT for oligometastatic disease, hopefully more patients will be diagnosed at an earlier stage of metastasis due to the higher detection rate. This accuracy in diagnosing metastasis in turn will allow for multidisciplinary treatment combining MDT.

### 3.3. Treatment

In the past, treatment of oligometastatic PC was discussed only in terms of ADT, which is the cornerstone of systemic treatment. However, in recent years, the significance of combined local treatment has been widely discussed; MDT may be useful in some cases and should be considered as part of multimodal therapy. The following mechanisms have been postulated: suppression of growth factors and immunosuppressive cytokines by eradicating the primary tumor site [91], reduction of circulating tumor cells [92], and promotion of the anticancer immune response by the abscopal effect (i.e., regression of metastases after therapeutic irradiation of the primary tumor site) [93]. However, there is no standardized treatment for the choice of local treatment because the superiority of RT over surgery is unknown due to the lack of direct comparative studies. Although there is no standardized treatment, there have been a few recent reports suggesting the efficacy of local RT. In 2018, a randomized controlled trial, the HORRAD trial, compared ADT with local radiation versus ADT alone for PC complicated by bone metastases [10]. Although the two groups had no significant difference in OS (HR: 0.90; 95% CI: 0.70–1.14; *p* = 0.4), a subanalysis suggested that RT to the prostate improved OS in patients with low tumor volume (<5 bone lesions). In 2018, the STAMPEDE trial investigated ADT ± docetaxel with local irradiation [6]; this was a phase 3 randomized controlled trial in which patients with newly metastatic PC were randomized 1:1 to receive ADT ± docetaxel as standard therapy with or without concomitant RT to the prostate. RT consisted of either daily irradiation (55 Gy/20 doses) for four weeks or weekly irradiation (36 Gy/6 doses) for six weeks. Although survival was significantly prolonged in the standard treatment plus RT group (HR: 0.76, 95% CI: 0.68–0.84; *p* < 0.0001), OS was not prolonged in the standard treatment plus RT group (HR: 0.92, 95% CI: 0.80–1.06; *p* = 0.266). Notably, in the subanalysis by tumor volume, there was a significant difference in OS at three years in the low metastatic volume group (73% in the standard treatment group vs. 81% in the standard treatment plus radiation group; HR: 0.68, 95% CI: 0.52–0.90; *p* = 0.007), whereas combined radiation treatment had no prognostic effect in the high metastatic volume group. In fact, the results of the HORRAD and STAMPEDE trials suggest the usefulness of local irradiation, since the combination of hormonal therapies with local RT was found to prolong prognosis in low metastatic burden [6,10]. However, the options for RT are diversifying, and it is currently unclear which irradiation method is optimal. Radiobiologically, the α/β value of PC is as low as 1.5 Gy, therefore RT for PC is considered to be more effective with higher doses in smaller fractions [42,43]. In summary, the efficacy of HDR brachytherapy, which delivers a high dose per instance of irradiation, has been recognized for the control of the primary disease; however, there are no reports of HDR brachytherapy against PC with oligometastasis.

These results suggest that the combined local irradiation is effective for patients with low metastatic volume. The CHAARTED study defines high metastatic volume as the presence of visceral metastases or ≥4 bone lesions with ≥1 beyond the vertebral bodies and pelvis [94], whereas low metastatic volume is often defined as having <3 bone metastases, which is exactly in line with the definition of oligometastasis. Furthermore, a few randomized controlled trials are currently underway to test the efficacy of local treatment for metastatic PC [95]. The SWOG 1802 trial (NCT01751438) is evaluating the effect of local treatment (external beam radiation or surgery) in addition to systemic treatment for M1 PC, while the g-RAMPP trial (NCT02454543) is evaluating the effect of RP plus extended lymph node dissection in addition to systemic treatment for M1b PC (≤5 bone metastases). These studies are expected to evaluate the efficacy of local treatment, and future results will be interesting.

As for systemic treatment, ADT is the basic treatment for locally advanced PC. Docetaxel and abiraterone, which is a new anti-androgenic agent, are both currently being used upfront, and the choice of these agents is also noteworthy. However, considering that the CHAARTED study, which demonstrated the benefit of early docetaxel administration in patients with untreated metastatic PC, was unable to show the survival benefit of docetaxel uptake in patients with low metastatic volume [94], the use of early chemotherapy or novel ADT for oligometastatic PC should be further investigated.

Treatment of metastases consists of surgical resection or SBRT. The goals may be to control the cancer, slow down further metastasis, and avoid or delay systemic therapy-related toxicity. A systematic review has given an overview of the current evidence for MDT in oligometastatic PC [11]. Local control rates at two years ranged between 76–100%. Progression free survival (PFS), its definition was inconsistent, was reported from 38–100% at one year and 22–83% at two years. The ORIOLE Trial, a randomized phase 2 study comparing observation and MDT, showed a significantly better PFS of MDT than observation (median, not reached vs. 5.8 months; HR: 0.30; 95% CI: 0.11–0.81; *p* = 0.002) [12]. Moreover, an interim analysis of a large prospective trial involving SBRT in oligometastatic PC patients with up to five metastatic sites was reported [13]. The proportion of patients who did not require treatment escalation (e.g., modification of ADT, introduction of chemotherapy, or palliative RT) was 51.7% (95% CI: 44.1–59.3%) at two years after SBRT, and the median survival without treatment escalation over the entire follow-up period was 27.1 months (95% CI: 21.8–29.4 months). In addition, PSA reduction was observed in 75% of patients. Therefore, the SBRT shows very promising potential for the long-term suppression of oligometastatic PC.

## 4. Conclusions

Although there is still no standard treatment for very high-risk locally advanced or oligometastatic PC, a major shift in treatment strategy is underway, with reports on the benefits of combined local RT with ADT as a cornerstone. On the other hand, RT delivery methods and protocols vary from study to study, which is an issue that remains unresolved. It will be interesting to see the results of currently ongoing large-scale studies in this field which will be reported in the future.

## Figures and Tables

**Table 1:** Definition of very high-risk prostate cancer.

Source (Year)	Definition	Reference
Spahn (2010)	2 high-risk features (PSA > 20 ng/mL, GS 8–10, and cT3–4)	[16]
Walz (2011)		[17]
Sundi (2014)	Primary Gleason pattern 5 or 4 cores containing pattern 4	[19]
Joniau (2015)	GS 8–10 in combination with 1 other high-risk factor (PSA > 20 ng/mL and cT3–4)	[18]
NCCN guidelines (2019v2)	cT3b–4 or primary Gleason pattern 5 or >4 cores with Grade Group 4 or 5	[20]
EAU guidelines (2019)	cT3–4 or cN+, any PSA, any GS	[23]

PSA = Prostate specific antigen; GS = Gleason score; NCCN = National Comprehensive Cancer Network; EAU = European Association of Urology.

**Table 2:** Summary of the treatment of high-risk localized and very high-risk prostate cancer.

Study	No. of Patients	Eligibility Criteria	Treatment	Median Follow-Up	Outcome	Reference
Radical prostatectomy for patients presenting with locally advanced disease						
Moltzahn	266	cT3b–4, N0 or N1, M0	RP + PLND ± adjuvant ADT and/or RT	9.3 years	10-year CSS 87.1–94.4%	[29]
External beam radiotherapy for patients presenting with locally advanced disease						
RTOG 85-31	977	cT3 or N1 or RP + PSM and/or SVI	RT vs. RT + ADT (lifelong)	11 years	10-year OS 39% vs. 49%, *p* = 0.002; 10-year CSS 78% vs. 84%, *p* = 0.005; 10-year MFS 61% vs. 76%, *p* < 0.0001	[3,4]
Intergroup randomized study	1205	cT3–4, N0/Nx, M0 or cT1–2 with either PSA > 40 ng/mL or PSA 20–40 ng/mL and GS 8–10	ADT (lifelong) vs. ADT + RT	8 years (range, 0–15.2)	10-year OS 49% vs. 55% (HR 0.70, 95% CI 0.57–0.85, *p* < 0.001); CSS higher in combined treatment (HR 0.46, 95% CI 0.34–0.61, *p* < 0.001); 10-year PFS 46% vs. 74% (HR 0.31, 95% CI 0.25–0.39)	[5]
STAMPEDE	638/2962	Two of the following are met; cT3–4, GS 8–10, PSA > 40 ng/mL	SOC vs. SOC + ZA vs. SOC + DOC vs. SOC + ZA + DOC	3.6 years (IQR 2.5–5)	5-year OS 55% vs. 57% vs. 63% vs. 60%; SOC + DOC (HR 0.78, 95% CI 0.66–0.93, *p* = 0.006) and SOC + DOC + ZA (HR 0.82, 95% CI 0.69–0.97, *p* = 0.022) compared to SOC only, respectively	[6]
GETUG 12	413	cT3–4 or GS 8–10 or PSA > 20 ng/mL or pN1	ADT (3 years) + local treatment + CT (DOC + EMT) vs. ADT + local treatment	8.8 years (IQR 8.1–9.7)	8-year RFS 62% vs. 50% (adjusted HR 0.71, 95% CI 0.54–0.94, *p* = 0.017)	[7]
Rusthoven	796	cT1–4, N1, M0	RT vs. no local treatment	5.2 years	10-year OS 45% vs. 29% (HR 0.58, 95% CI 0.48–0.71, *p* < 0.001); 10-year CSS 67% vs. 53% (HR 0.61, 95% CI 0.47–0.80, *p* < 0.001)	[8]
Lin	3540	cN1, M0 or Mx	ADT + RT vs. ADT alone	5.2 years	5-year OS 71.5% vs. 53.2% (HR 0.50, 95% CI 0.37–0.67, *p* < 0.001)	[9]
Various modalities of radiotherapy						
Zelefsky	296	cT3, N0, M0	3D-CRT or IMRT+ ADT (3 months)	8 years (range, 2–16)	10-year RFS T3a/T3b: 44%/32%; 10-year CSS T3a/T3b: 88%/79%	[44]
Mizowaki	120	cT3–4, N0, M0	IMRT + ADT (6 months)	97 months (range, 21–120)	8-year RFS 53.2%; 8-year CSS 96.6%; 8-year OS 89.1%	[51]
Goupy	276	cT3b, N0–1, M0	IMRT + ADT (3 years)	26 months (95% CI, 33–39)	5-year RFS 75.2%; 5-year CSS 89.7%; 5-year OS 78.8%	[53]
Yamazaki	249	cT3b–4, N0, M0	HDR + EBRT vs. HDEBRT vs. Conv EBRT	64 months (range, 13–153)	5-year RFS 78.9% vs. 88.1% vs. 66.5% (*p* = 0.0003)	[73]
Kishan	1809	Gleason score 9–10	RP vs. EBRT vs. EBRT + LDR/HDR	RP, 4.2 years; EBRT 5.1 years; EBRT + LDR/HDR 6.3 years	5-year MFS 76% vs. 76% vs. 92% (*p* < 0.001); 7.5-year OS 83% vs. 82% vs. 90% (*p* < 0.05)	[75]

RP = radical prostatectomy; PLND = pelvic lymph node dissection; ADT = androgen deprivation therapy; RT = radiotherapy; SOC = standard of care; ZA = zoledronic acid; DOC = docetaxel; IQR = inter-quartile range; HR = hazard ratio; CI = confidence interval; OS = overall survival; CSS = cancer-specific survival; MFS = metastasis-free survival; PFS = progression-free survival; RFS = recurrence-free survival; CT = chemotherapy; EMT = estramustine; 3D-CRT = three-dimensional conformal radiation therapy; IMRT = intensity modulated radiation therapy; HDR = high-dose-rate; LDR = low-dose-rate; HD = high dose; Conv = conventional; EBRT = external beam radiotherapy.

**Table 3:** Definition of oligometastatic prostate cancer.

Source	No. of Patients	Number of Metastases	Number of Metastases	Detection	Reference
Tabata	35	5	Bone only; each site < 50% size of vertebral body	Bone scan	[79]
Ahmed	17	5	NS	^11^C-choline PET–CT, MRI, biopsy, CT	[80]
Berkovic	24	3	Bone or LN	Bone scan, ^18^F-FDG PET–CT, ^11^C-choline PET–CT	[81]
Schick	50	4	NS	Bone scan, ^18^F-choline PET–CT, ^11^C-acetate PET–CT	[82]
Decaestecker	50	3	Bone or LN	^18^F-FDG PET–CT, ^18^F-choline PET–CT	[83]
Ost	119	3	Any	^18^F-FDG PET–CT, ^18^F-choline PET–CT	[84]

CT = computed tomography; MRI = magnetic resonance imaging; PET = positron emission tomography; FDG = fluorodeoxyglucose; LN = lymph node; NS = not specified.
